# Identification of isoliquiritigenin as an activator that stimulates the enzymatic production of glycyrrhetinic acid monoglucuronide

**DOI:** 10.1038/s41598-017-10154-y

**Published:** 2017-10-02

**Authors:** Xiaoxue Wang, Dong Wang, Yixin Huo, Dazhang Dai, Chihua Li, Chun Li, Guiyan Liu

**Affiliations:** 1 School of Life Science, Beijing Institute of Technology, 5 South Zhongguancun Street, Haidian District, Beijing 100081 P.R. China; 2 Mailman School of Public Health, Columbia University, New York City, USA

## Abstract

Glycyrrhetinic acid monoglucuronide (GAMG) is a great value-added and has considerable commercial interest due to its strong pharmacological activities and functional low-calorie sweetener. However GAMG is quite rare in natural plants, and it must be prepared from glycyrrhizin (GL) by hydrolysing one terminal glucuronic acid. *β*-Glucuronidase is the key enzyme in the biotransformation of GL to GAMG, but its activities need to be enhanced to facilitate the industrial large-scale production of GAMG. In this study, we identified that isoliquiritigenin (ISL), as one of chemical compositions from the total flavonoids glycyrrhiza (TFG), can significantly enhance *β*-glucuronidase activity *in vitro*. Measurements using high-performance liquid chromatography (HPLC) showed that the activity of *β*-glucuronidase could be increased by 2.66-fold via the addition of ISL to a *β*-glucuronidase solution that contained GL at a 3:10 molar ratio of ISL to GL. ISL was concluded to be an activator because ISL could reduce the K_m_ and E_a_ of *β*-glucuronidase reacting with GL. This study sheds new light on the mechanism of *β*-glucuronidase and helps to make industrial production of GAMG through fermentation feasible.

## Introduction

Glycyrrhizic acid (GL) is a major active ingredient in the herb medicine *Glycyrrhiza* ^1^, but its bioavailability is low. In the human body, GL takes effects via being transformed into glycyrrhetinic acid monoglucuronide (GAMG) and glycyrrhetinic acid (GA) by intestinal microorganisms^2^. Compared with GL, GAMG has strongeranti-tumour, anti-allergy and anti-inflammatory pharmacological activities, and is easily permeates the cell membrane because of its weaker polarity^3,4^. GAMG also serves as an artificial functional low-calorie sweetener; its sweetness is 941-fold greater than that of sucrose, and 5-fold more than that of GL^5,6^. However, GAMG is quite rare in natural plants and only can be produced by biosynthesis or hydrolysis of GL. It is very significant, therefore, to transform GL to GAMG with high efficiency.

**Figure 1:**
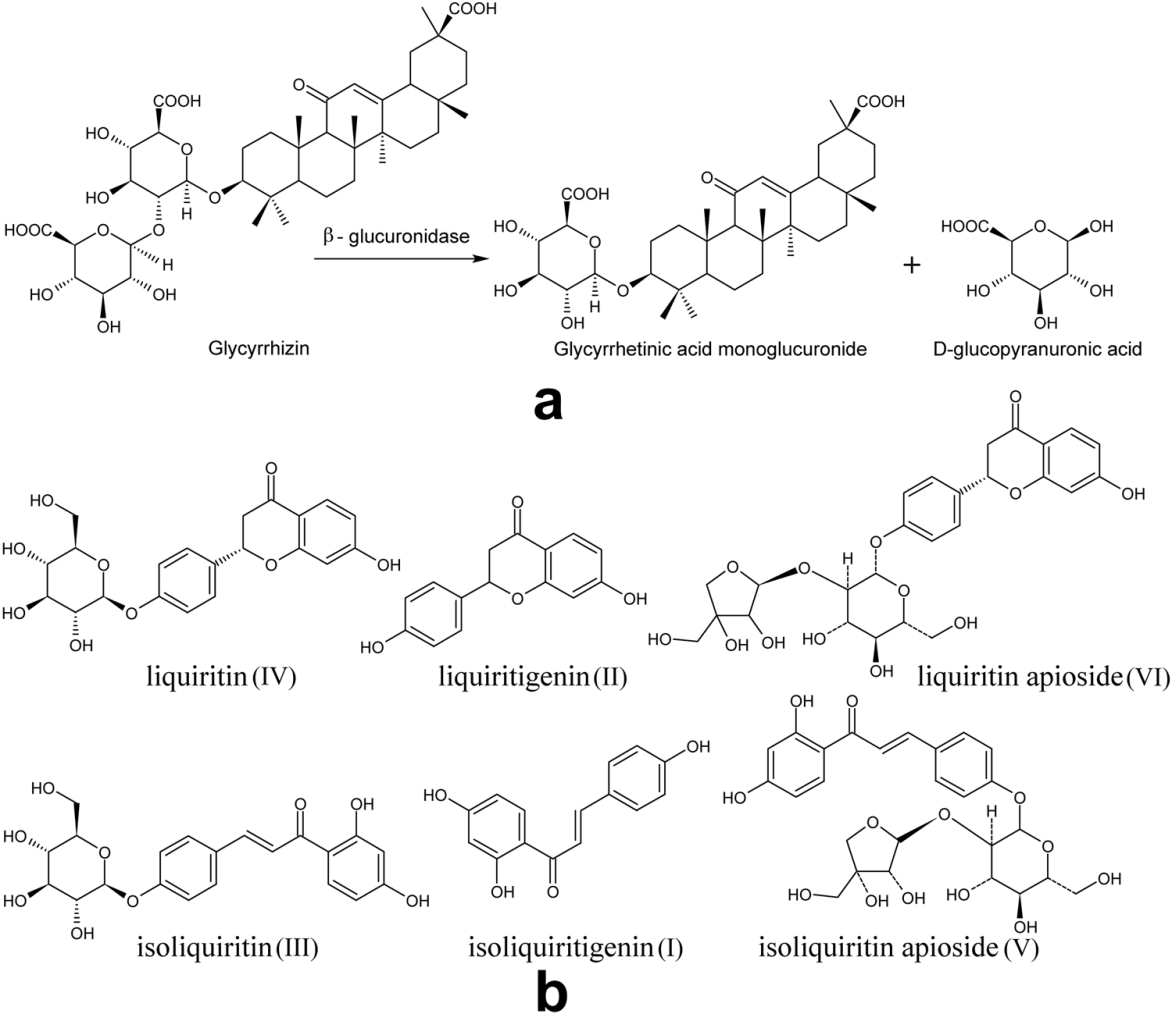
Reactions and structures. (**a**) Hydrolysis reaction of GL to GAMG catalyzed by *β*-glucuronidase. (**b**) The structures of the six compounds separated from TFG.

Because of the similar reactivity of the O-glycosidic bond for the two glucuronides, it is not easy to directionally synthesize GAMG from GL by chemical methods. As an eco-friendly method with high specificity, mild reaction conditions and catalytic efficiency, the biotransformation of GAMG from GL is a preferred method (Fig. 1a). So far, all the *β*-glucuronidases that can convert GL to GAMG derive from enterobacteria^2,7^, fungi^8–10^ and animal organisms^11^, which are limited by the low selectivity of the O-glycosidic bond, weak enzymatic activity and high production cost, respectively. Therefore, the industrial conversion of GL to GAMG is still in an early stage and is not a major contributor for the growing market. Previously, we reported that the fungus *Penicillium purpurogenum* Li-3 can produce *β*-glucuronidase with high chemical bond selectivity^10^. When grown in liquid media, it hydrolyzed GL to GAMG directly. However, the conversion ratio of GL was only 88.45% after 96 h of fermentation, indicating that the amount and activities of the enzymes were relatively low. Therefore, it is essential to figure out how to increase the enzyme activity of *β*-glucuronidase and reduce the production cost of GAMG.

GL is abundant in liquorice, accounting for 2% of the total weight^12^. The isolate of GL from liquorice is a multistep process. We speculated that the production cost of GAMG could be reduced by adding *P. purpurogenum* Li-3 directly to the total extract of glycyrrhiza (TEG). However, we could not find any GAMG despite TEG having a high content of GL (7.3%). Interestingly, the activity of *β*-glucuronidase increased about threefold when an appropriate amount of TEG was added to a culture medium that contained GL as its carbon source^13^ (mass ratio of TEG to GL was 1:1).

In order to explore this phenomenon, we have done many studies in this paper. We separated the total flavonoids of glycyrrhiza (TFG), the total triterpenoids of glycyrrhiza (TTG), and the total saccharides of glycyrrhiza (TSG) from TEG and tested the effects of each component on the whole-cell enzymatic activity of *β*-glucuronidase from *P. purpurogenum*Li-3 by adding them into the media containing GL. The components that increased the enzymatic activities were further isolated and analysed via adding them into enzyme solution that contained the GL to determine the effective compound. The results indicated that isoliquiritigenin (ISL) greatly enhances the conversion of GL, which could potentially reduce the raw material cost during the fermentation process. We concluded that the ISL in the TFG enhances *β*-glucuronidase activity as an activator. Furthermore, the mechanism of the high activity of the β-glucuronidase was explored. Michaelis-Menten model calculations indicated that ISL promotes the affinity of *β*-glucuronidase for the substrate GL. The *K*_*m*_ value decreased significantly compared to the control, whereas the *V*_*max*_ increased. A calculation based on the Arrhenius equation showed that ISL decreased the activation energy by 6.31%compared to the control, which indicated that adding ISL could effectively stabilize the enzyme-substrate transition state. The research flow chart is shown in Supplementary Fig. S1. The novelty of this report is that we identify ISL as the activator of *β*-glucuronidase, which has a promising future for the bioconversion GL to GAMG. Our work may also shed new light on the industrial production of GAMG.

## Results

### pH-dependent separation of active ingredients

Studies have focused on the efficient separation of the active ingredients from *Glycyrrhiza*. Fu *et al*.^14^ separated liquorice flavonoids and glycyrrhizic acid by using macroporous resins. However, the final purity was only 66% and the extraction was time-consuming. Sun *et al*. and Shen *et al*.^15,16^ studied the separation of glycyrrhizic acid and liquiritin by aqueous two-phase extraction with a non-ionic surfactant and three-liquid-phase extraction systems, respectively. However, the non-ionic surfactant that was used, Triton X-100, was costly, and the procedure of the latter method was complex. In our study, we found that pH had a significant effect on the extraction of the three components of *Glycyrrhiza*. Extraction by changing the pH of the solution twice offers the highest separation capacity for TFG, TSG and TTG with high extraction recovery, procedural simplicity and low cost. Moreover, the recovery (97%) is 5% higher than previously reported for other methods^17^. In addition, our method is patented^18^.

**Figure 2:**
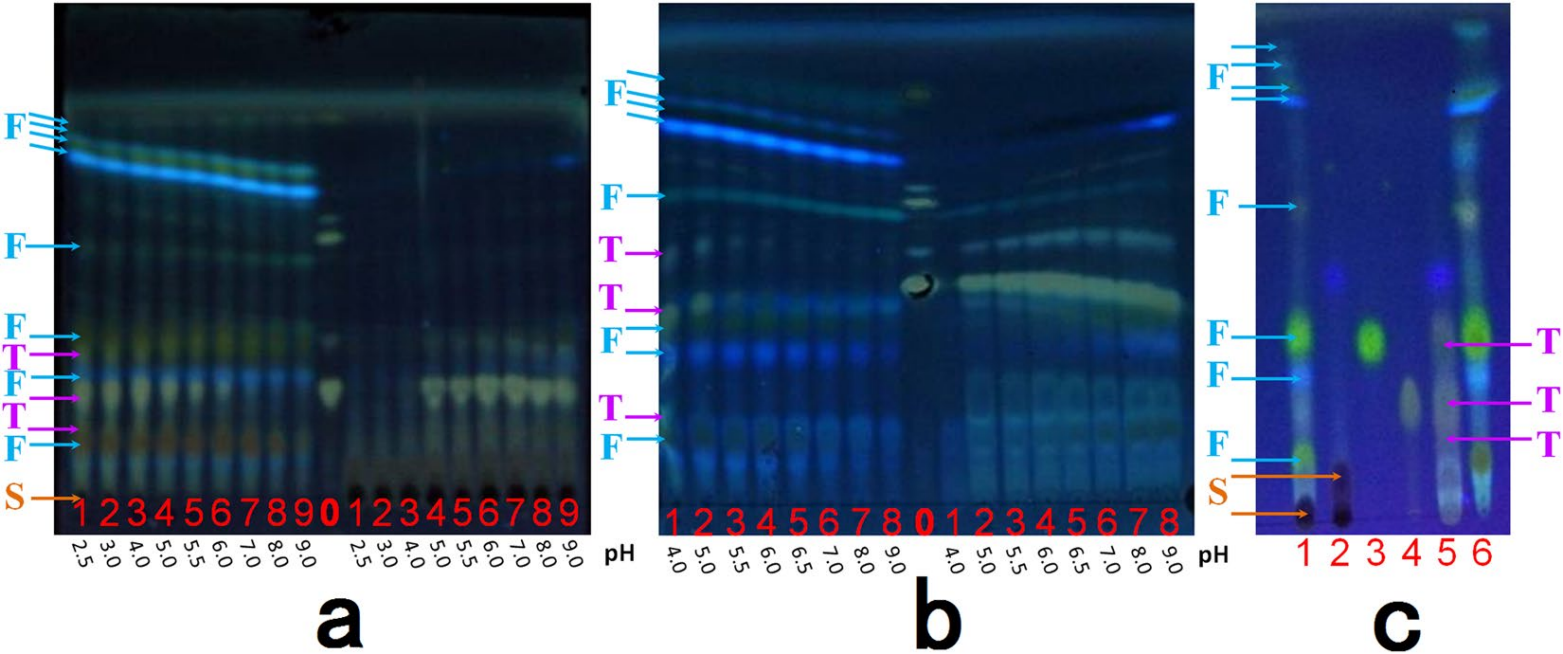
TLC of the TTG, TFG and TSG adapt to pH change and the results of separation. (0: the control of mixture of GA GAMG and GL, S: saccharide, T: triterpenoid, F: flavonoids: left is organic phase, right is aqueous phase. (**a**) TTG, TFG and TSG adapt to pH change. (**b**) TTG and TFG adapt to pH change. (**c**) The results of separation).

As shown in Fig. 2a, pH had a great influence on TTG. Concentration of TTG that was in the organic decreased with the increase of the pH of the solution. When pH ≤ 4.0, TTG and all TFG was in the organic phase and changed little, but almost all TSG was in the aqueous phase. After separating the water layer, the organic layer was used to further determine how to separate TFG and TTG.

The effect of solution pH on the extraction of TFG and TTG is shown in Fig. 2b. The amount of TFG that was in the aqueous phase increased, and the amount of TTG in the organic phase decreased with an increase in the pH of the solution. When pH = 7.0, nearly all the TFG is in the organic phase, and the TTG was only detected in the aqueous phase. Thus, TFG and TTG can be effectively separated. The result of the separation of TFG, TTG and TSG is shown in Fig. 2c.

### Effect of TTG, TFG and TSG on the production of *β*-glucuronidase enzymes in *P. purpurogenum* Li-3

**Figure 3:**
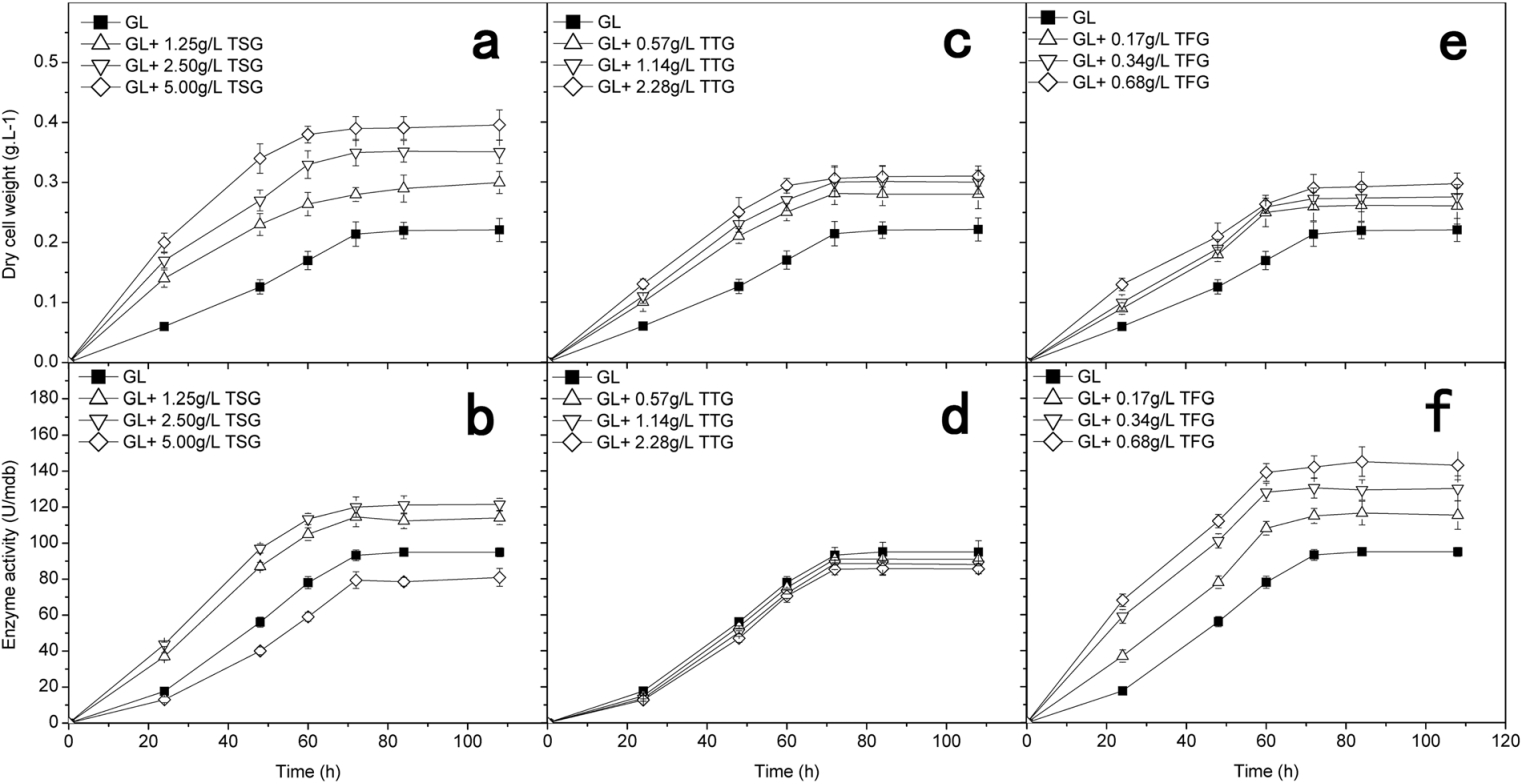
Effects of the three compounds on the biomass and specific activity of *β*-glucuronidase (n = 3).

As shown in Fig. 3a, the biomass of the *P.purpurogenum* Li-3 in the absence of TSG in the control group reached 0.22 mg/mL after 108 h of cultivation. The biomass of the identical strain in the presence of 0.5, 1, and 2 times of TSG (1 time of a fraction is equal to content of TSG in 4 g/L TEG, namely TSG is 2.50 g/L) in the experimental group were 0.30 (+/−0.018), 0.34 (+/−0.019), and 0.38 (+/−0.025) after 108 h of cultivation, respectively. The results indicated that TSG could promote the growth of *P. purpurogenum* Li-3 (the composition of various medium are seen in strains cultivation method).

The whole-cell enzyme activity of *β*-glucuronidase that was produced in the absence of TSG in the control group was 94 U/mg dry biomass (mdb). The specific whole-cell enzyme activity of *β*-glucuronidase from whole-cell that was produced in the presence of 0.5 and 1 times of TSG in the experiment group increased were to 112 U/mdb and 120 U/mdb, respectively. The specific enzyme activity of *β*-glucuronidase that was produced in the presence of 2 times of TSG in the experimental group decreased to 80 U/mdb (Fig. 3b). These results indicated that TSG could affect the specific enzyme activity of *β*-glucuronidase in a dose-dependent pattern in the fermentation culture medium containing BCM, GL and TSG.

This dose-dependent pattern was mainly attributed to the availability of the carbon sources. Proper amount of TSG can increase the growth of strains and then increase the amount of enzyme produced. The saccharides in TSG were consumed easily by *P. purpurogenum* Li-3 because these saccharides were mainly aldose that rather than uronic acid. When aldose was consumed, the strains have to consume GL that is the only carbon source. The GL will not be easy utilized if the culture media contained too much aldose. Therefore, it was limited that GL Induced *P. purpurogenum* Li-3 production of *β*-glucuronidase.

As shown in Fig. 3c, the biomass of *P. purpurogenum Li-3* in the absence of TTG in the control group reached 0.22 mg/ml after 108 h of cultivation. The biomass of the identical strain in the presence of 0.5, 1, and 2 times of TTG (1 time of a fraction is equal to content of TTG in 4 g/L TEG, namely TTG is 1.14 g/L) in the experimental group were 0.28 (+/−0.024), 0.29 (+/−0.020), and 0.30 (+/−0.017) mg/ml, respectively. The results indicated that TTG could increase the growth of *P. purpurogenum* Li-3.

The whole-cell enzymatic activity of *β*-glucuronidase that was produced in the absence of TTG in the control group was 94 U/mdb. The specific whole-cell enzyme activity of *β*-glucuronidase from whole-cell that was produced in the presence of 0.5, 1, and 2 times of TTG in the experimental group decreased to 91, 88 and 85 U/mdb, respectively (Fig. 3d). The results indicated that TTG could inhibit slightly the enzyme activities of *β*-glucuronidase in a dose-dependent pattern in the fermentation culture media containing BCM, GL and TTG. This pattern could be explained by the existence of inhibitors in TTG, which can inhibit the enzyme activity of *β*-glucuronidase.

As shown in Fig. 3e, the biomass of the *P. purpurogenum* Li-3 in the absence of TFG in the control group reached 0.22 mg/ml after 108 hr of cultivation. The biomass of the identical strain in the presence of 0.5, 1, and 2 times of TFG (1 time of a fraction is equal to content of TFG in 4 g/L TEG, namely TFG is 0.34 g/L) in the experimental group were 0.26 (+/−0.030), 0.28 (+/−0.021), and 0.30 (+/−0.018) mg/ml, respectively. The results indicated that TFG could increase the growth of *P. purpurogenum* Li-3.

The whole-cell enzyme activity of *β*-glucuronidase that was produced in the absence of TFG in the control group was 94 U/mdb. The specific whole-cell enzyme activity of *β*-glucuronidase that was produced in the presence of 0.5, 1, and 2 times of TFG in the experimental group increased to 116, 134 and 145 U/mdb, respectively (Fig. 3f). The results indicated that TFG could increase the specific enzyme activities of *β*-glucuronidase significantly in a dose-dependent pattern in the medium containing BCM, GL and TFG. Therefore, it was clear that the activation effect of TEG on the whole-cell enzyme activity came from TFG. Further studies were needed to investigate which component in TFG increased the enzyme activity of *β*-glucuronidase.

### Structural identification of flavonoids from Glycyrrhiza

In order to determine the exact chemical compositions that was enhancing the *β*-glucuronidase activity, six primary flavonoid compounds were isolated from the TFG, and their structures were identified by NMR and MS.

Compound I was obtained as yellow needle crystals (methanol), which appeared orange with the addition of 10% H_2_SO_4_. The^1^H-NMR (see Supplementary Fig. S2) spectrum showed a typical chalcone signal at δ 7.77 (1 H, d, J = 4.2 Hz) and 7.76 (1 H, d, J = 4.2 Hz). Three of the fifteen distinct signals in the^13^C-NMR (see Supplementary Fig. S3) spectrum were observed at δ 192.0 (C = O), 117.9 (C-α) and 144.7 (C-β), which also exhibited typical characteristics of the chalcone signal. In addition, the^1^H-NMR also showed ABX and AA ‘XX’ coupling patterns of two groups of aromatic protons; the former group was at δ 8.17 (1 H, d, J = 9.0 Hz), 6.42(1 H, dd, J = 9.0, 2.3 Hz), and 6.29 (1 H, dd, J = 2.3 Hz), and the other was at δ 7.76 (2 H, d, J = 8.3 Hz) and 6.83 (2 H, d, J = 8.3 Hz). The compound’s molecular formula of C_15_H_12_O_4_ was established from the molecular ion peak at m/z 256 [M]^+^ in the EI-MS. Since all of the above data were consistent with those of the reference^19^, compound I was identified as isoliquiritigenin.

Compound II was colourless needle crystals (methanol) and appeared yellow with the addition of 10% H_2_SO_4_. It is similar to compound I; the ABX and AA ‘XX’ coupling patterns of aromatic protons also appeared in the^1^H-NMR (Fig. S4) spectrum. In addition, the pattern of peaks at δ 5.44 (1 H, dd, J = 13.0, 2.8 Hz), 3.13(1 H, dd, J = 16.8, 13.0 Hz) and 2.63 (1 H, d, J = 16.8, 2.8 Hz) were assigned to the three protons at H-2 and H-3. The typical dihydrogen flavonoid signals appeared in the^13^C-NMRspectrum (see Supplementary Fig. S5) at δ 190.6(C-4), 79.4(C-2) and 43.6(C-3). The compound’s molecular formula of C_15_H_12_O_4_ was established from the molecular ion peak at m/z 256 [M]^+^ in the EI-MS. These data corresponded to those of the liquiritigenin in references^19^. Thus, it was identified as liquiritigenin.

Compound III was obtained as a yellow powder (methanol). Compared to compound 2, it showed an additional group of glucose signals in the^13^C-NMR (see Supplementary Fig. S7) spectrum at δ 100.7 (glc-l), 73.7 (glc-2), 77.5 (glc-3), 70.2 (glc-4), 77.0 (glc-5) and 61.1 (glc-6). Its molecular formula was established as C_21_H_22_O_9_ on the basis of the molecular ion peak at m/z 441 [M+Na]^+^ by ESI-MS analysis. It was identified as isoliquiritin^20^.

Compound IV was a white power (methanol). With the exception of having glucose features, its^13^C-NMR (see Supplementary Fig. S9) spectrum was the same as that of Compound 1. Its molecular formula was established as C_21_H_22_O_9_ on the basis of the molecular ion peak at m/z 441 [M+Na]^+^ in ESI-MS. It was identified as liquiritin^19^.

Compound V and VI were named isoliquiritin apioside and liquiritin apioside, respectively^21^.These name were given because their^13^C-NMR (see Supplementary Fig. S11, 13) spectra included a group of apiose signals thatcompounds4 and 3 did not have, and EI-MS indicated a molecular formula of C_26_H_30_O_13_ for both of them.

### Influence of the primary compounds from TFG on the *β*- glucuronidase activity

To validate the influences of the 6 primary compounds from TFG on the enzymatic activities of *β*-glucuronidase, the compounds were added respectively into the enzyme solutions containing the 2.4 mmol/LGL. As shown in Supplementary Table S1, only the addition of compound I (ISL) could increase the activity of *β*-glucuronidase, and the activation effect became more significant with the increase of the concentration of ISL. According to Table 1, the production rate of GAMG in the control group was 2.63 μmol/(L·min). When the addition amount of ISL was at molar concentration ratio 3: 10 of ISL to GL, the production rate of GAMG rose to 7.01 μmol/ (L.min), which is 2.66 times that in the control group. Further addition of ISL did not increase evidently.

**Table 1:** The relationship between concentration of ISL and reaction rate*(n = 3).

Item	Serial							
	0	1	2	3	4	5	6	7
Buffer (mL)	0.2	0.194	0.188	0.176	0.152	0.12	0.04	0
18 mmol/L ISL (mL)	0	0.006	0.012	0.024	0.048	0.08	0.16	0.2
18 mmol/L GL (mL)	0.2	0.2	0.2	0.2	0.2	0.2	0.2	0.2
*β*-D-glucuronidase solution (mL)	0.8	0.8	0.8	0.8	0.8	0.8	0.8	0.8
GAMG (mmol/L)	0.63 ± 0.02	0.79 ± 0.01	0.93 ± 0.02	1.2 ± 0.03	1.68 ± 0.04	1.71 ± 0.03	1.72 ± 0.01	1.71 ± 0.01
*v* (μmol/L·min)	2.63 ± 0.07	3.29 ± 0.04	3.86 ± 0.07	4.99 ± 0.1	7.01 ± 0.15	7.13 ± 0.13	7.16 ± 0.02	7.11 ± 0.03
ISL (mmol/L)	0	0.09	0.18	0.36	0.72	1.2	2.4	3

*v = c/t, where c is the concentration of GAMG, t is the reaction time. The reaction was conducted at 170 rpm and 40 °C for 4 h. The GL concentration of the control group is 2.4 mmol/L and no ISL added.

In previous studies, we attempted to enhance the enzyme activity of *β*-glucuronidase to optimize the reaction that was induced by *P. purpurogenum* Li-3 and to improve the enzyme expression level. For example, Song *et al*. integrated the *β*-glucuronidase gene into an E. coli BL21 (PGUS-E) expression system to enhance the expression level of PGUS-E. However, the selectivity of PGUS-E for the two O-glycosidic bonds of GL was weakened, producing a GA by product in the final GAMG product^22^. He *et al*. used anionic liquid to improve catalytic environments to enhance the chemical bond selectivity of PGUS-E to a level that is comparable to that of the wild-type fungus, but the high cost of the ionic liquid made it impractical for industrial production^23^. In this study, we showed that adding ISL is a simple but feasible method to increase the activity of *β*-glucuronidase.

### Effect of ISL on the reaction

**Figure 4:**
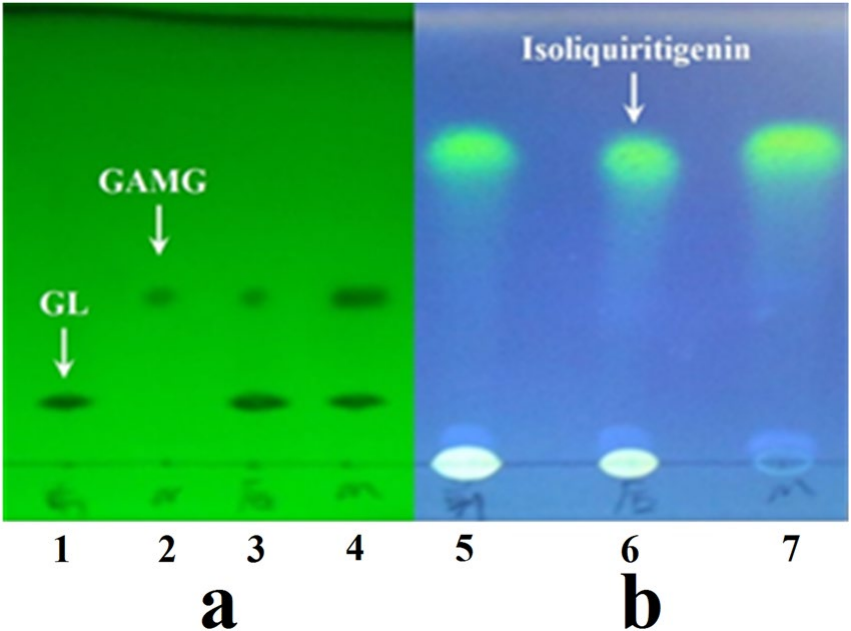
TLC results of effects of ISL on reaction (**a)** the picture of the 254 nm, developing solvent: chloroform-ethyl acetate-acetic acid-water (1.5: 4.0: 2.0: 1.0). (**b**) The picture of the 365 nm which treated by 10% sulfuric acid, developing solvent: chloroform-ethyl acetate-methanol (5.0: 4.0:0.4). 1. GL control; 2. GAMG control; 3. after transformation in GL reaction system; 4. after transformation in GL reaction system which added ISL; 5. before transformation in ISL system which added ISL; 6. after transformation in r ISL system which added ISL; 7. ISL control).

To determine further whether ISL is the substrate of *β*-glucuronidase, just like GL, the results of ISL before and after reaction were analyzed using thin-layer chromatography (TLC). ISL did not change into other ingredients after reaction and the concentration of ISL remained unchanged at the end of the reaction (Fig. 4b), indicating that ISL was not the reaction substrate of *β*-glucuronidase. In addition, the amount of GAMG in the reaction system that contained ISL was significantly larger than that in the control, indicating that ISL could greatly enhance the conversion rate (Fig. 4a). Hence, we concluded that the ISL in TFG could enhance *β*-glucuronidase activity as an activator.

### Effect of ISL on the expression of *β*-glucuronidase

Inducers and activators are the two common effectors capable of improving enzyme activity. For *β*-D-glucuronidase, GL was reported to act as an inducer^10^ in improving *β*-glucuronidase enzyme activity. However, it was still unknown how ISL improves the enzyme activity. Therefore, BCM + GL (GL as carbon source) + *P. purpurogenum* Li-3 was set as experimental group 1, and BCM + ISL (ISL as carbon source) + *P. purpurogenum* Li-3 was set as experimental group 2. The mycelia were collected after 72 h of fermentation. We broke the cells and extracted the enzyme protein for SDS-PAGE analysis^24^.

**Figure 5:**
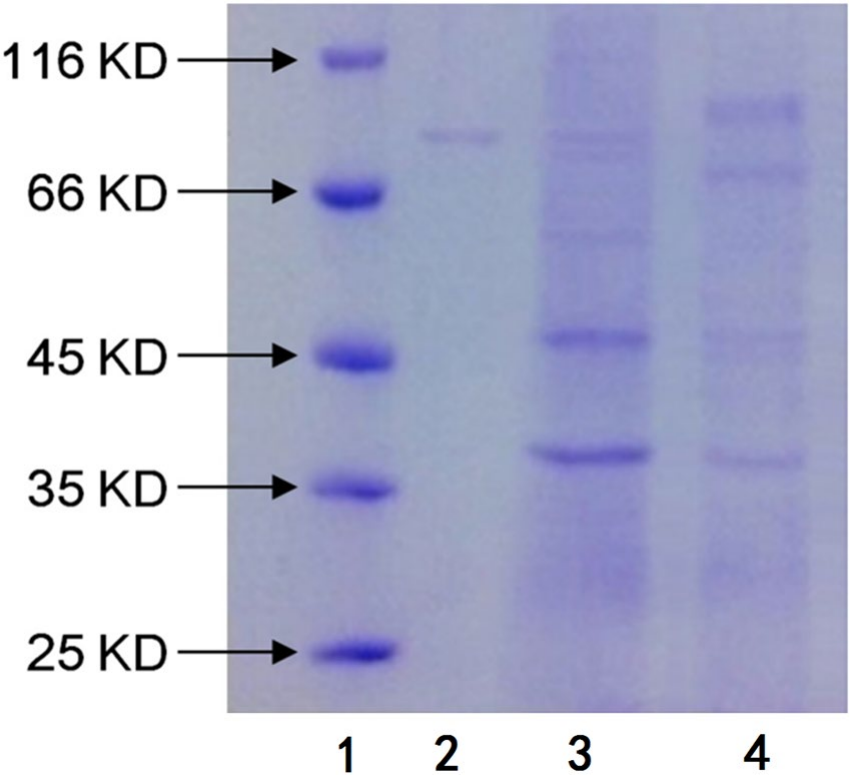
SDS-PAGE of *β*-glucuronidase produced from *P. purpurogenum* Li-3. (1. Maker; 2. *β*-glucuronidase; 3. the experimental group Containing GL; 4. the experimental group Containing ISL).

From the results shown in Fig. 5, ISL did not induce *P. purpurogenum* Li-3 to produce *β*-glucuronidase, which suggested that ISL was not an inducer. Therefore, we came to the conclusion that ISL was an activator of *β*-glucuronidase. Currently, the majority of studies on enzyme activators are on medicines^25–27^, with few on sweeteners. In our study, ISL was first presented as an activator of *β*-glucuronidase, not only providing an innovative method for the production of GAMG but also enriching the application of enzyme activators in the field of food science.

### Influence of ISL on the reaction kinetic parameter and apparent activation energy

**Figure 6:**
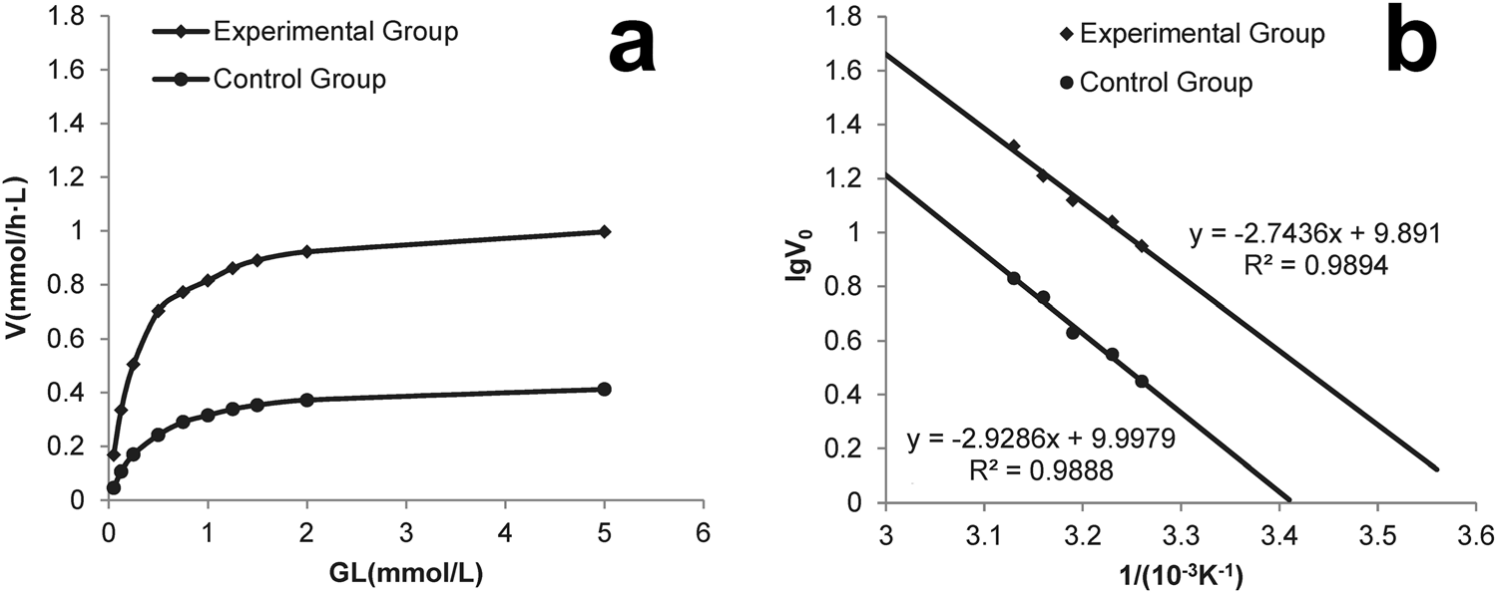
Michaelis-Menton plots of the promotion kinetics and Arrhenius plot method calculated the apparent activation energy of β-glucuronidase when added ISL. (**a)** Control group: GL, Experimental group: GL + ISL, 2 h reaction at 40 °C in crude β-glucuronidase solution. (**b**) Control group: GL; Experimental group: GL + ISL).

Using to the double reciprocal treatment of the Michaelis-Menten model (Fig. 6a), the kinetic parameters of GL and GL supplemented with isoliquiritigenin (1 mmol/L) could be calculated (Table 2). These results indicated that ISL promoted the affinity of *β*-glucuronidase for GL since the *K*_*m*_ value decreased significantly compared to the control; in contrast, the *V*_*max*_ increased.

**Table 2:** Apparent kinetic parameters and activation energy of the hydrolysis of GL catalyzed by *β*-glucuronidase.

System	*K*_*m*_ (mmol)	*V*_*max*_ (mmol·h^−1^)	*V*_*max*_/*K*_*m*_ (h)	*E*_*a*_ (kJ·mol^−1^)
Experiment	0.26	1.05	4	52.53
Control	0.41	0.45	1.1	56.07

The Arrhenius law, which is based on empirical observations, is commonly used for estimating the relationship between reaction rate constants and temperature^28^. To explain the increased reaction rate, we studied the reaction rate o f *β*-glucuronidase in reaction systems that had and did not have ISL (control). The apparent activation energy for the catalysis of GL was calculated (Fig. 6b, Table 3) using the Arrhenius equation. The results showed that ISL decreased the activation energy by 6.31%, which indicated that adding ISL could effectively stabilize the transition state of the enzyme-substrate complex and result in the higher catalytic activity of the enzyme.

## Discussion

In this study, we determined that ISL as an activator to stimulates the enzymatic production of glycyrrhetinic acid monoglucuronide. Then how does ISL work? We explored the mechanism of its action as follows.

The *β*-glucuronidase from *P. purpurogenum* Li-3 is an apparent 69.72 KDa dimer with an unknown crystal structure at present^29^; thus, we can only infer the mechanism of the ISL activation effect on the *β*-glucuronidase. After the formation of an enzyme-substrate complex (ES) from the substrate and enzyme, only a fraction of the ES could be converted to the proper product (P). Part of the ES could reversibly and conformationally change to form an inactive enzyme-substrate complex (ES2) that induces substrate inhibition effects on enzyme activity. Previously, Pesheck, P. *et al*.^30^ observed that t-butanol could increase the activity of *β*-glucuronidase by 9-fold. Although t-butanol could increase the V_max_ of the conversion from ES to P, the activation of *β*-glucuronidase by t-butanol is mainly due to a decrease in the inactive form ES2, which reduces the substrate inhibition effect. Solvents such as chloroform exhibit paradoxical activation effect on the activities of *β*-glucuronidases from bacteria and mammalian cells. Although chloroform, as a protein-denaturing agent, has strong denaturing and harmful effects on both bacteria and mammalian *β*-glucuronidases, the addition of chloroform in the reaction system could significantly increase the conversion rate of the corresponding *β*-glucuronidase-dependent reaction. Therefore, apparently, chloroform is an activator of *β*-glucuronidase. Michael T. proved that the mechanism of this paradoxical effect is that chloroform could serve as an emulsifier to increase the surface area of the liquid-liquid interface^31^. As an emulsifier, chloroform increased the collisions and interactions between *β*-glucuronidase and substrates, eventually increasing the substrate conversion rate and other activity parameters.

In our study, the TFG have an activation effect on *β*-glucuronidase. We further isolated six compounds from the TFG. Among them, ISL, as a chalcone, has the lowest polarity and could be categorized as a water repellent compound. Therefore, we hypothesized that ISL might function as an activator through both mechanisms. First, as a non-polar compound, ISL is likely to be able to bind *β*-glucuronidase and stabilize the conformation of *β*-glucuronidase. This interaction could increase the formation of active ES, reduce the conversion from active ES to inactive ES2, and eventually increase the conversion from ES to P. Other isolated compounds have no such effect due to the steric hindrance. Second, ISL, with similar character as organic solvents, has the potential to increase the specific surface area of the contact between *β*-glucuronidase and its substrate, therefore increasing the rate of substrate conversion to its corresponding products by *β*-glucuronidase. Taken together, in this study, we found that ISL, a compound that was isolated from the TEG, is a very efficient activator of the enzyme *β*-glucuronidase. ISL could greatly enhance the conversion of GL, which could potentially reduce raw material costs during the fermentation process.

Although the dry weight of the TTG is 17% of the TEG and GL is the major compound of the TTG (40%), the conversion of the raw material GL to GAMG by *P. purpurogenum* Li-3 fermentation is limited by the low activity of *β*-glucuronidase. To identify the activator of *β*-glucuronidase to potentially decrease the industrial production cost, we added a variety of compounds that were isolated from the TEG into the *in vivo* fermentation or *in vitro* enzymatic assay systems. We concluded that ISL could enhance the enzymatic activities of PGUS. ISL remained unmodified and could be utilized repeatedly. Therefore, the total cost of the production process could be potentially significantly reduced because the steps to purify GL from the TEG could be removed. This study sheds new light on the mechanism of *β*-glucuronidase and helps to make the industrial production of GAMG through fermentation feasible.

## Methods

### Materials and reagents

The *P. purpurogenum* Li-3that was used in this work was preserved in the China General Microbiological Culture Collection Center (CGMCCNo.5446) for patent protection in China. The *Glycyrrhiza uralensis* Fisch was purchased from the Xinjiang Tianshan Pharmaceuticals Industry Co., Ltd., and the glycyrrhizin (purity ≥ 98%) was purchased from the Tongtian Biotech Co., Ltd. The liquiritin, isoliquiritin, liquiritigenin, isoliquiritigenin, liquiritin apioside, and isoliquiritin apioside were self-purified to purities ≥ 98%. The *β*-glucuronidase that was used was purified using our previously reported method^29^. Other common analytical grade reagents were purchased commercially.

### Preparation of TEG

100 g of rhizomes of *Glycyrrhiza uralensis* Fisch was cut into slices approximately 3 mm thick and mixed with deionized water three times (1 h for each time). The V: M ratios of deionized water and medical materials were 10, 8 and 6 for the first, second, and third time, respectively. The three extractions were mixed, vacuum and dried to produce 25 g of TEG that contained 7.3% GL.

### Separation of TFG, TTG and TSG

25 g of TEG was dissolved in 250 ml of deionized water. In total, 90 mL of the solution was divided into 9 groups, and 10 ml of organic solution was added to each group and mixed by a magnetic stirrer. For each group, the pH was adjusted by acid and base to the desired pH value. After mixing, 10 ml of solution was transferred into a separatory funnel. The organic and aqueous phases were analysed by thin-layer chromatography (TLC) to determine the suitable extraction pH value. Then, the extraction solution was obtained to determine the conditions for reverse extraction. After separation, 80 ml of the solution was drawn from the organic layer and divided into 8 groups. For each group, 10 ml of double-distilled water was added, and the solutions were analysed by the method described above.

### Preparation of the TFG, TTG, and TSG

15 g of TEG was dissolved in 150 ml of deionized water. The pH was adjusted to the desired value. (pH adjusted to 4), followed by the addition of 150 ml of organic solution. A total of 8.34 g of TSG was obtained from the solution in the lower layer. The top layer (the organic solution) was transferred to a new separatory funnel. The solution was adjusted again to desired pH value, and mixed with 150 ml of organic solution. A total of 1.26 g of TFG was isolated from the organic solution in the secondary top layer, and 3.99 g of TTG was isolated from the lower layer. The ratio of TFG:TTG: TSG was 3:10:22.

### Isolation of flavonoid compounds

The TEG was extracted three times from 5 kg of the dried roots of *Glycyrrhiza uralensis* Fisch with ethanol. The extraction solution was vaporized in vacuo and then suspended in water. The pH was adjusted to the desired value. After adding organic solution, 112 g of TFG was obtained from the organic solution in the top layer. Then, two compounds were isolated from each of the three fractions by Sephadex-LH-20 and C18 chromatography. These six compounds (compounds I-VI) were identified using thin-layer chromatography (TLC), high-performance liquid chromatography (HPLC), NMR and MS to be isoliquiritigenin, liquiritigenin, isoliquiritin, liquiritin, isoliquiritin apioside, and liquiritin apioside, respectively (Fig. 1b).

### Strains cultivation method

Base culture medium (BCM) consisted of base inorganic salts3.00 g/LNaNO3, 0.50 g/L K2HPO4, 0.50 g/L MgSO4•7H2O, 0.50 g/L KCl, and 0.01 g/L FeSO4•7H2O. The seed culture medium contained of BCM and glucose (5 g/L). The Enzyme produced culture medium that was optimized for enzyme production contained of BCM and GL (4 g/L). Fermentation culture media consisted of BCM and different dosages of TFG, TTG, and TSG. Culture medium and containers were sterilized at 118 °C for 15 min. The *P. purpurogenum* Li-3 was grown in a seed culture medium (100 ml) at 170 rpm and 30 °C for 72 h. The fungal mycelia were inoculated into different types culture medium and were cultured at 170 rpm and 30 °C for 120 h.

### Measurement of whole-cell enzyme activity

The whole-cell enzyme activity was measured as previously reported^32,33^. At the end of fermentation, 100 ml of individual fermentation broth was centrifuged, and the supernatant was removed. The fungal strain were washed with sodium acetate buffer (pH 5.0) three times and then mixed with 100 ml of 4 g/L GL solution at 170 rpm and 37 °C for 12 h. The concentration of GAMG was analysed by HPLC. One activity unit (U) of the whole-cell enzyme was defined to be the amount of enzyme (whole-cell biomass) that releases1 μmol of GAMG per hour under certain conditions. The specific activity (U/mg) was defined as the enzyme activity units that were exhibited by 1 mg of dry fungal strain under certain conditions.

### The specific activities of whole-cell enzymes in the presence of the TFG, TTG and TSG

Since TEG was a complex mixture, it was separated into three fractions (TSG, TFG and TTG). We added 0.5, 1, and 2 times of each fraction (1 time of a fraction is equal to its content in 4 g/L TEG), into the BCM + GL + *P. purpurogenum* Li-3. The biomass and specific activity of *β*-glucuronidase were measured every 12 h after the first 24 h of cultivation.

### Preparation of enzyme solution of *β*-glucuronidase

We prepared the enzyme solution by liquid nitrogen freezing and grinding with mortar and pestle^34,35^. The fungal mycelia of *P. purpurogenum* Li-3, which were collected by centrifugation at the end of the fermentation, were washed with 0.1 M sodium acetate buffer (pH 5.0) three times. The samples were then frozen in liquid nitrogen and ground to a fine powder for 20 min in a prefrozen mortar and pestle with a small amount of quartz sand. The powder was transferred to 0.1 M sodium acetate buffer (pH 5.0) and centrifuged, and the supernatant was collected as the enzyme solution of *β*-glucuronidase. The *β*-glucuronidase was obtained by ammonium sulfate fractionation and DEAE-cellulose chromatography.

### Measurement of *β*-glucuronidase enzyme activity

In total, 400 μl of the *β*-glucuronidase enzyme was added to 200 μl of sodium acetate buffer (pH 5.0) that contained 2.4 mg/ml GL. After shaking in a water bath at 40 °C for 4 h, the enzyme was heat-inactivated. Then, 100 μl of the mixture was diluted to 1 ml with methanol, and the concentration of GAMG in the solution was measured by HPLC. One activity unit (U) of the *β*-glucuronidase enzyme was defined as the amount of enzyme capable of releasing 1 μmol of GAMG per minute under the above conditions.

### HPLC analysis

The concentrations of GL and GAMG were measured with HPLC using an ODS column (Shim-pack, VP-ODS, 4.6 mm × 250 mm, Shimadzu Corporation, Kyoto, Japan) and a UV detector. The wavelength was set at 254 nm. A flow rate of 1.0 ml/min and an injection volume of 10 μl were used. The mobile phase was a mixture of 0.6% (v/v) acetic acid (pH 2.85) and methanol at a ratio of 19:81 (v/v). The retention times of GL and GAMG were approximately 7.5 and 15.9 min, respectively.

### Statistical analysis

Results are presented as the mean ± S.D. and analysed by the analysis of variance. All tests were two-sided with P < 0.05 indicating significant differences.

## Electronic supplementary material

Supplementary Information
