# Supplementary material for: Identification of isoliquiritigenin as an activator that stimulates the enzymatic production of glycyrrhetinic acid monoglucuronide
Source: Sci Rep. 2017 Oct 2;7:12503. doi: 10.1038/s41598-017-10154-y (PMC5624897; doi:10.1038/s41598-017-10154-y)
Supplement: Supplementary file 1 — Supplementary Information [file 41598_2017_10154_MOESM1_ESM.pdf]

Identification of isoliquiritigenin as an activator that stimulates the enzymatic production of glycyrrhetinic acid monoglucuronide

Xiaoxue Wang[1], Dong Wang[1], Yixin Huo[1], Dazhang Dai[1], Chihua Li[2], Chun Li[1], Guiyan Liu[1,*]

[1]School of Life Science, Beijing Institute of Technology, 5 South Zhongguancun Street, Haidian District, Beijing100081, P.R. China

[2] Mailman School of Public Health, Columbia University, New York City, U. S. A

[*]gyliu@bit.edu.cn

Contents

Figure S 1. **The research flow chart**

Figure S 2. **^{1}HNMR spectrum of Isoliquiritigenin in DMSO**

Figure S 3. **^{13}CNMR spectrum of Isoliquiritigenin in DMSO**

Figure S 4. **^{1}HNMR spectrum of Liquiritigenin in DMSO**

Figure S 5. **^{13}CNMR spectrum of Liquiritigenin in DMSO**

Figure S 6. **^{1}HNMR spectrum of Isoliquiritin in DMSO**

Figure S 7. **^{13}CNMR spectrum of Isoliquiritin in DMSO**

Figure S 8. **^{1}HNMR spectrum of Liquiritin in DMSO**

Figure S 9. **^{13}CNMR spectrum of Liquiritin in DMSO**

Figure S 10. **^{1}HNMR spectrum of Isoliquiritin apioside in DMSO**

Figure S 11. **^{13}CNMR spectrum of Isoliquiritin apioside in DMSO**

Figure S 12. **^{1}HNMR spectrum of Liquiritin apioside in DMSO**

Figure S 13. **^{13}CNMR spectrum of Liquiritin apioside in DMSO**

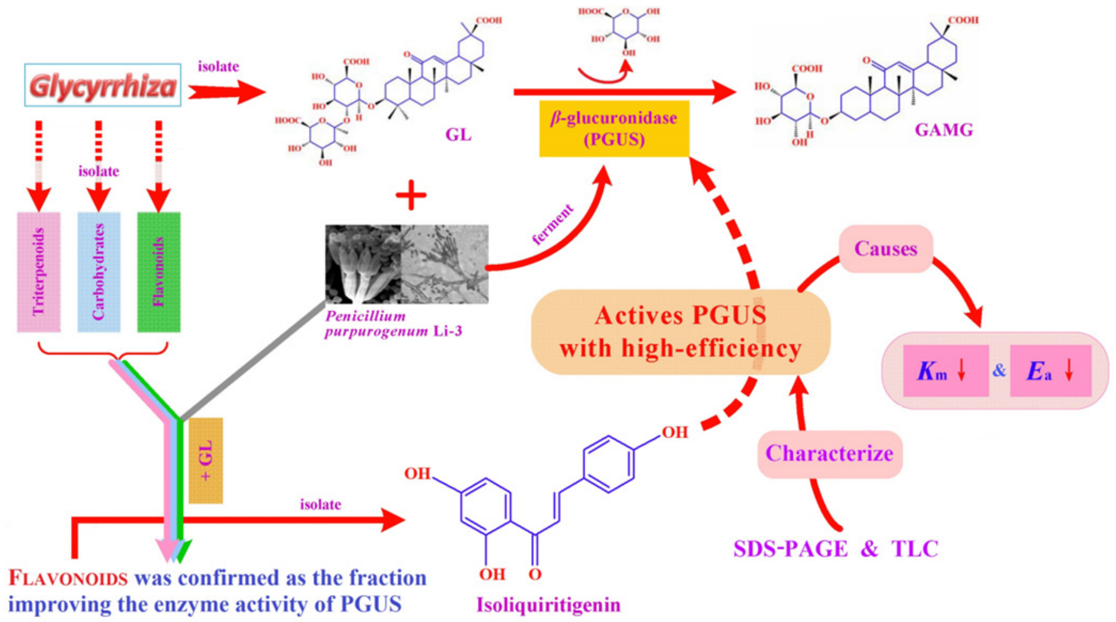

Figure S 1. The research flow chart

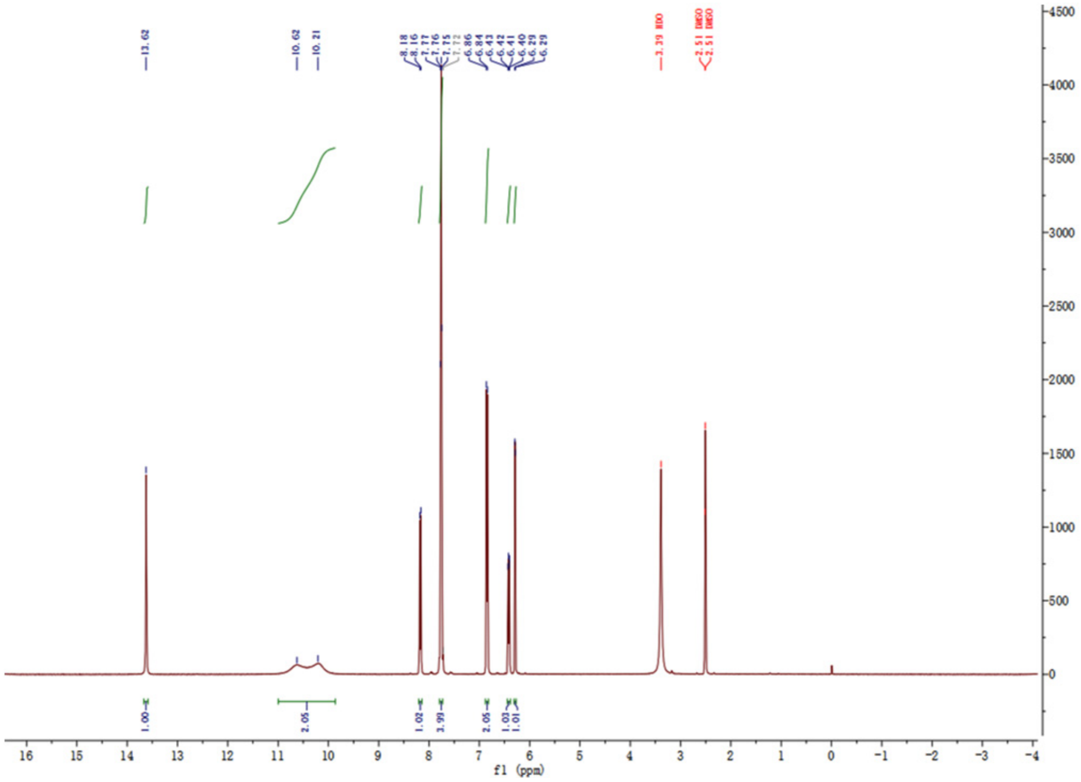

Figure S 2. ^{1}HNMR spectrum of Isoliquiritigenin in DMSO

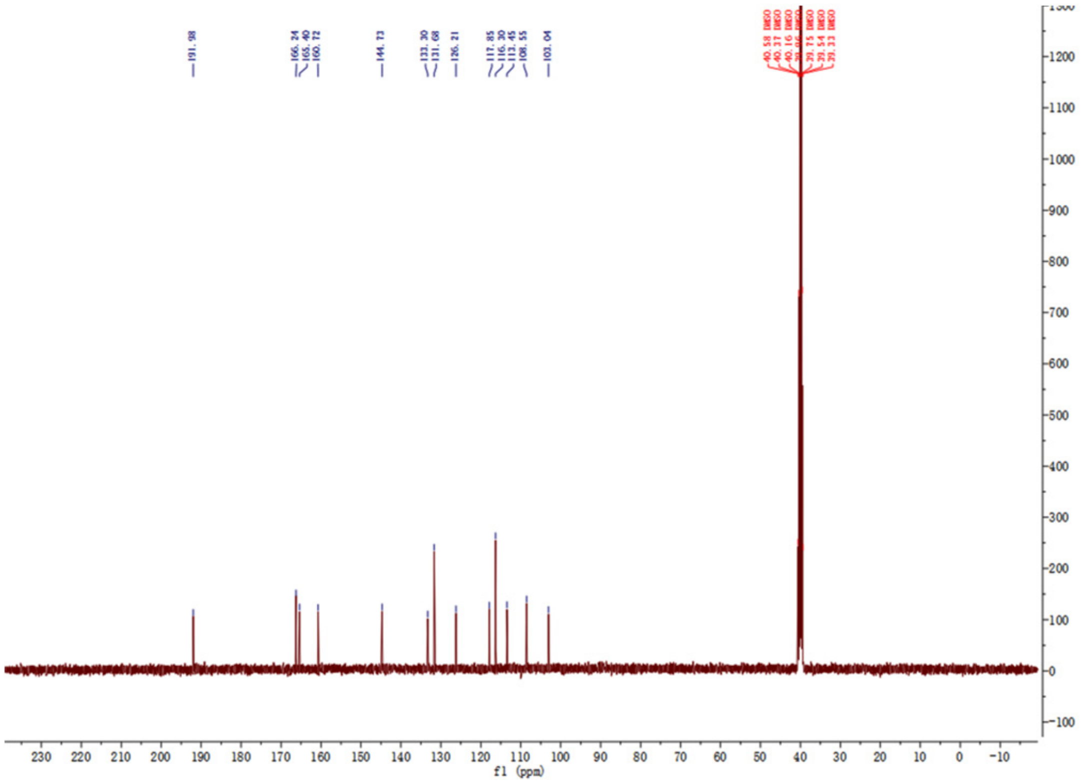

Figure S 3. ^{13}CNMR spectrum of Isoliquiritigenin in DMSO

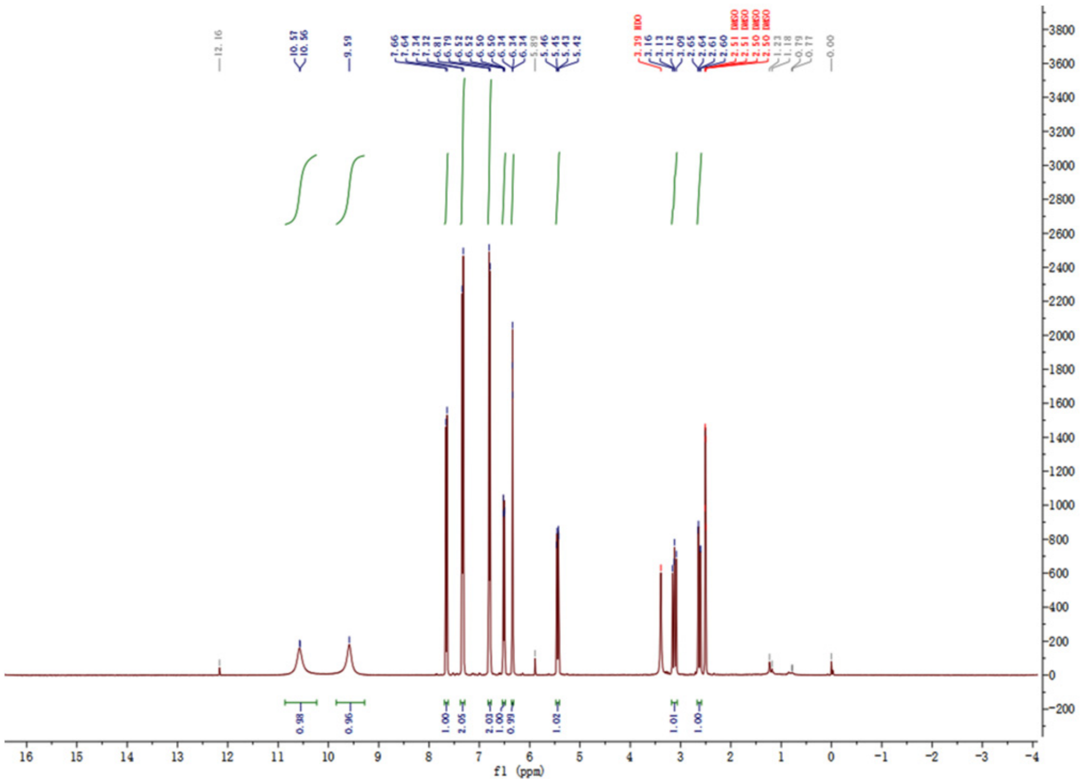

Figure S 4. ^{1}HNMR spectrum of Liquiritigenin in DMSO

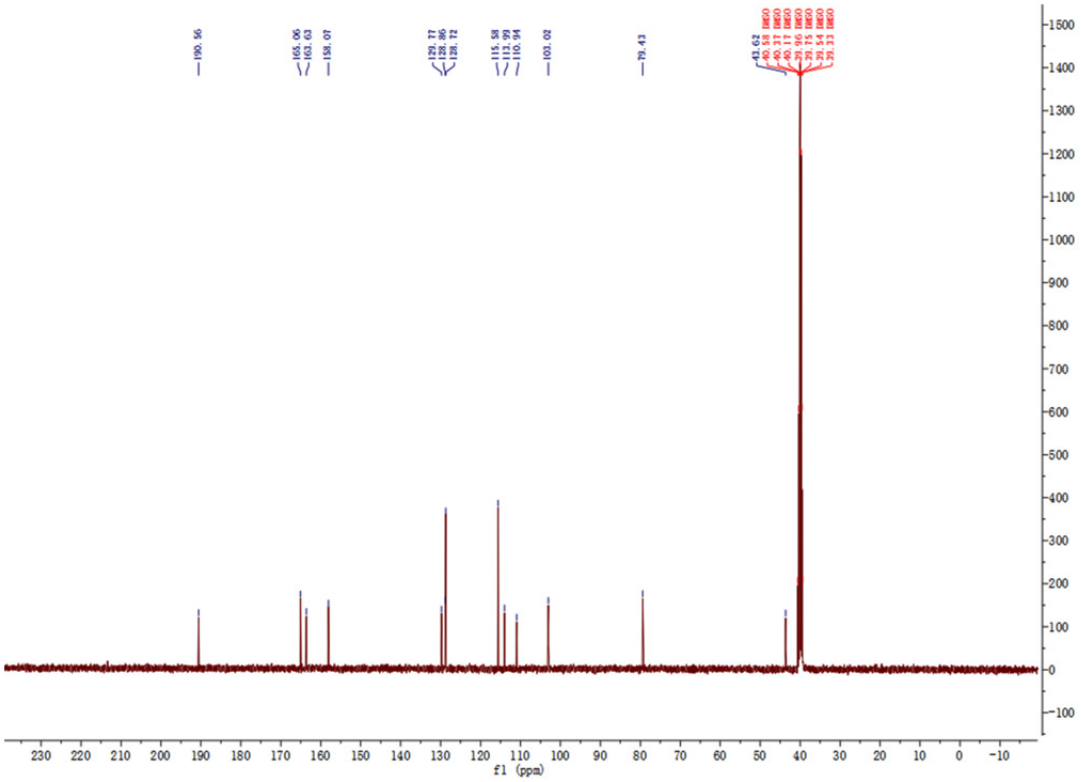

Figure S 5. ^{13}**CNMR spectrum of Liquiritigenin in DMSO**

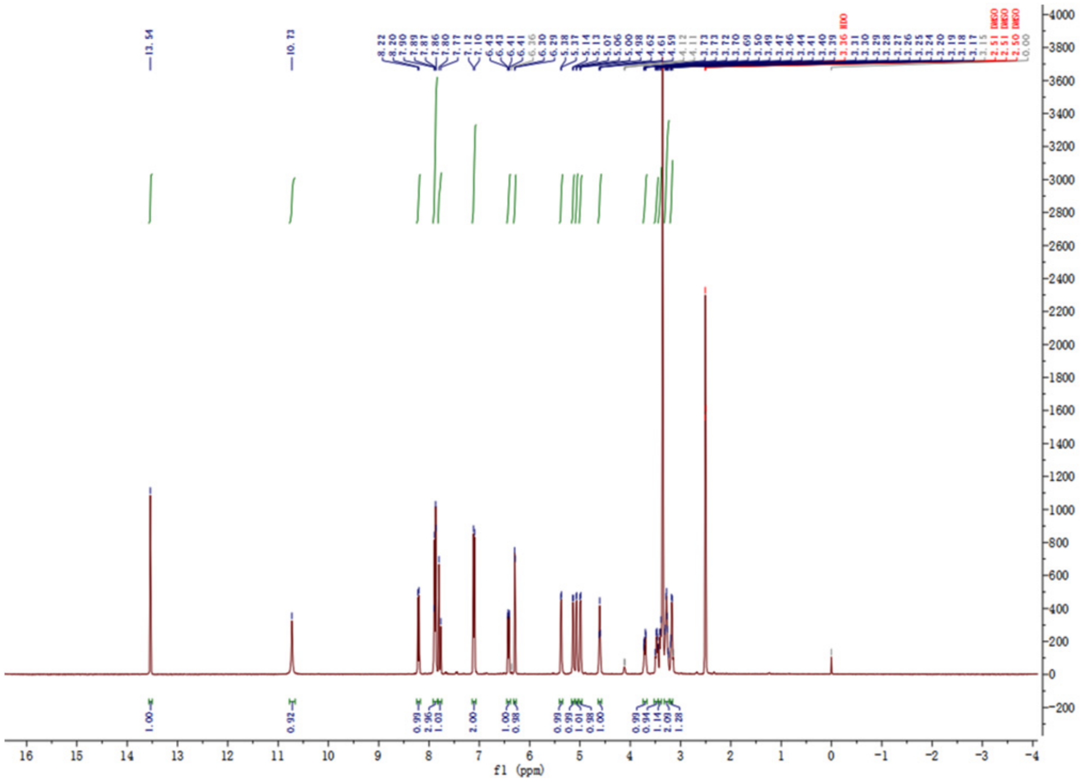

Figure S 6. ^{1}**HNMR spectrum of Isoliquiritin in DMSO**

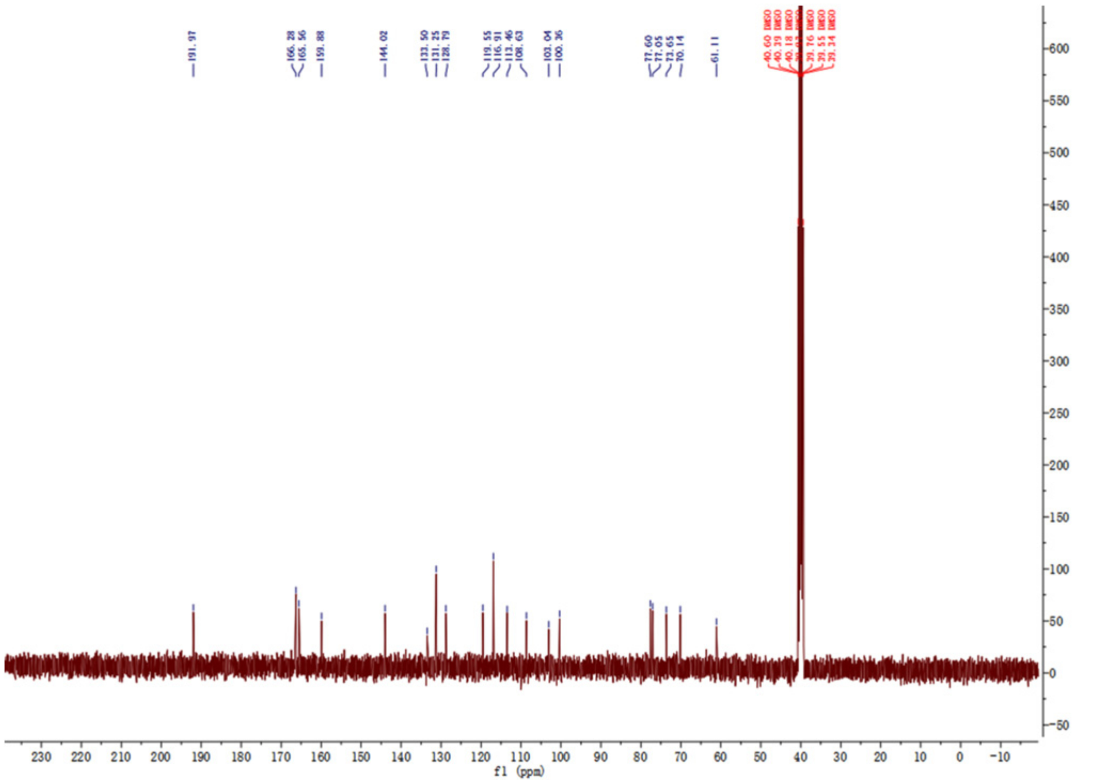

Figure S 7. ^{13}CNMR spectrum of Isoliquiritin in DMSO

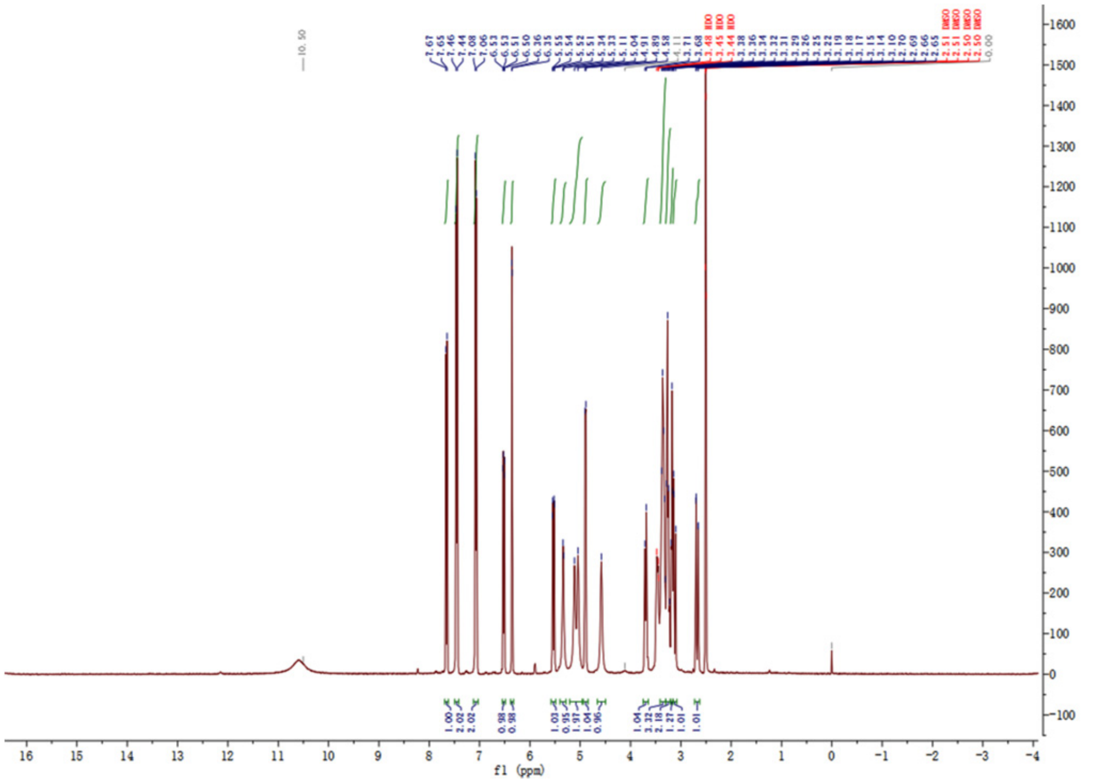

Figure S 8. ^{1}HNMR spectrum of Liquiritin in DMSO

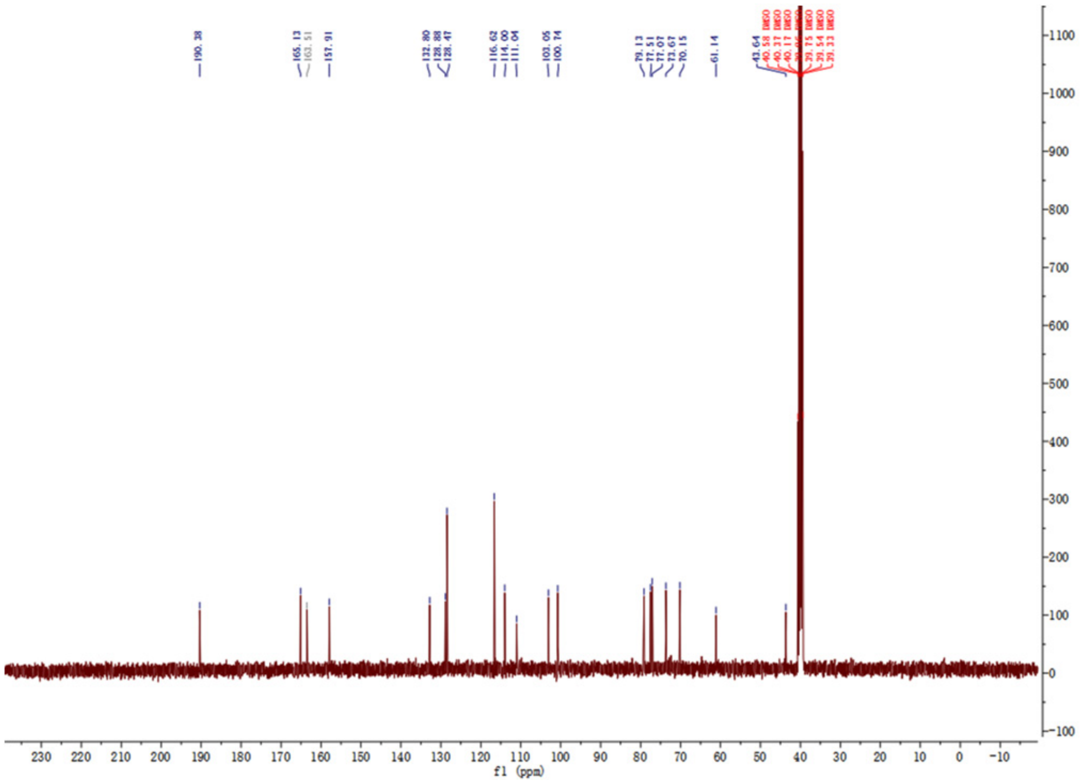

Figure S 9. ^{13}**CNMR spectrum of Liquiritin in DMSO**

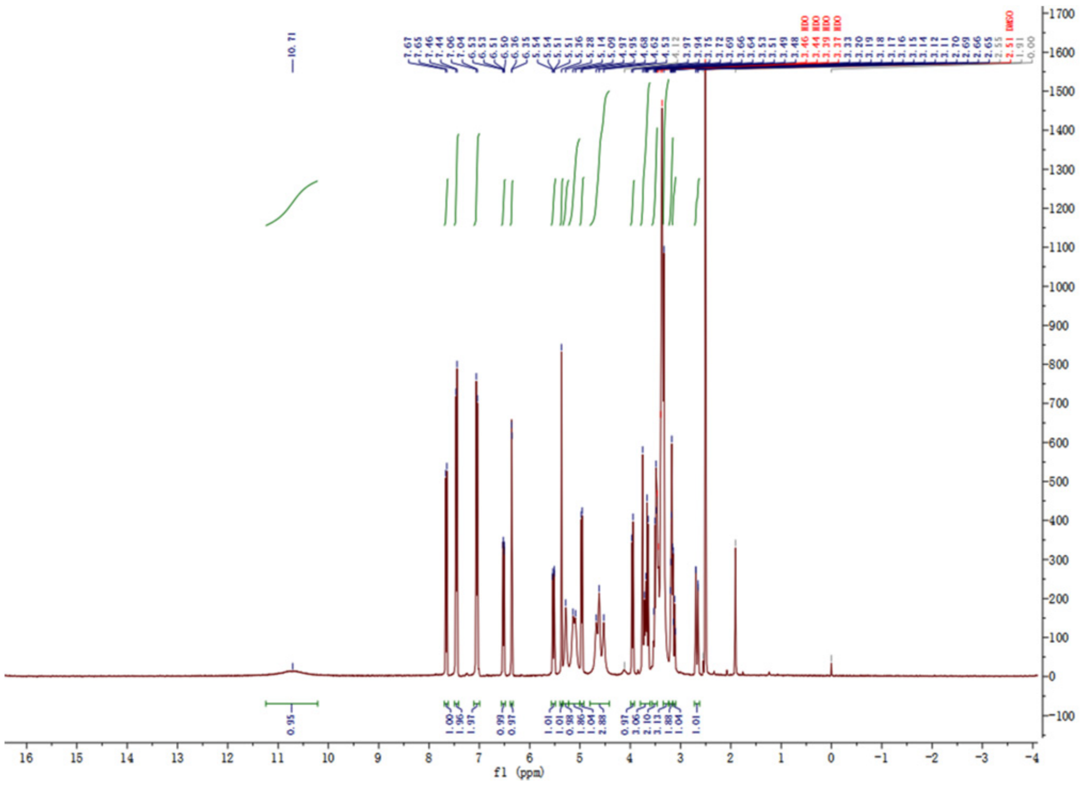

Figure S 10. ^{1}**HNMR spectrum of Isoliquiritin apioside in DMSO**

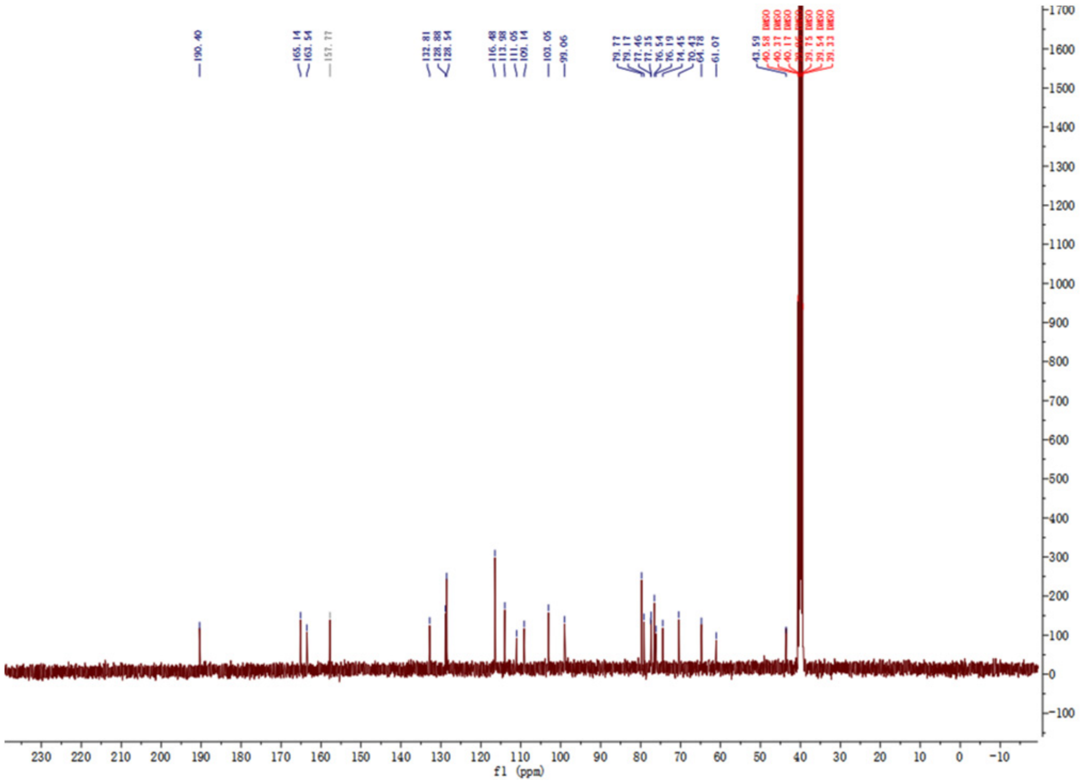

Figure S 11. ^{13}**CNMR spectrum of Isoliquiritin apioside in DMSO**

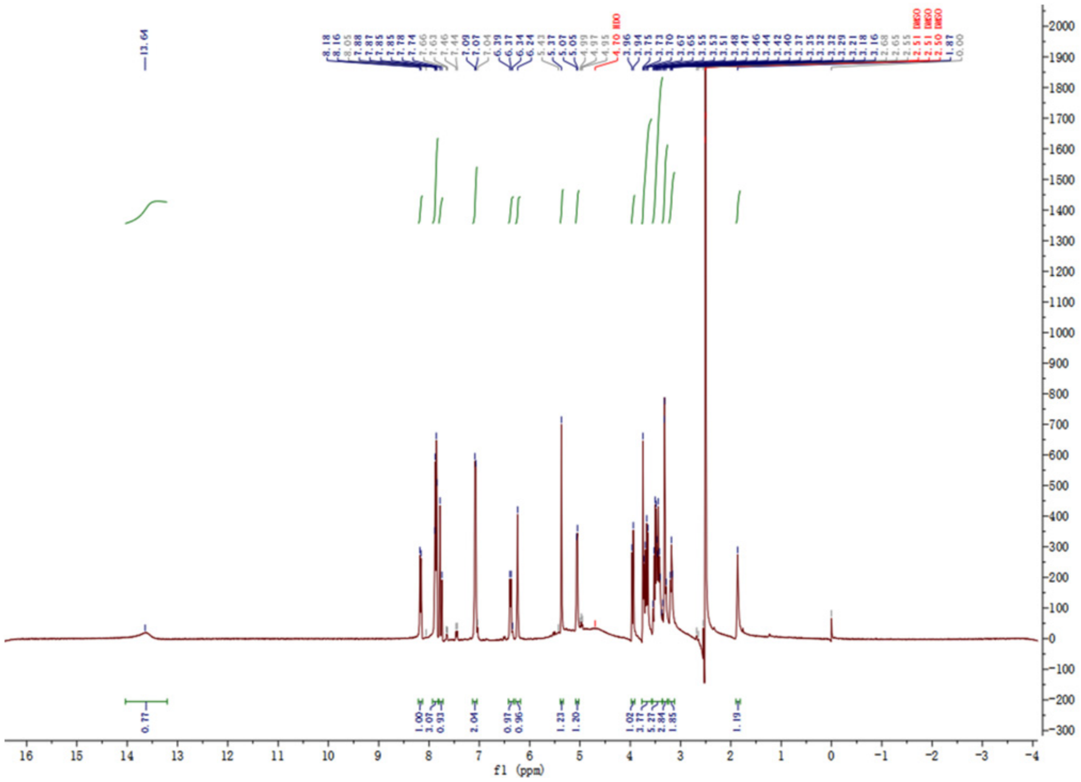

Figure S 12. ^{1}**HNMR spectrum of Liquiritin apioside in DMSO**

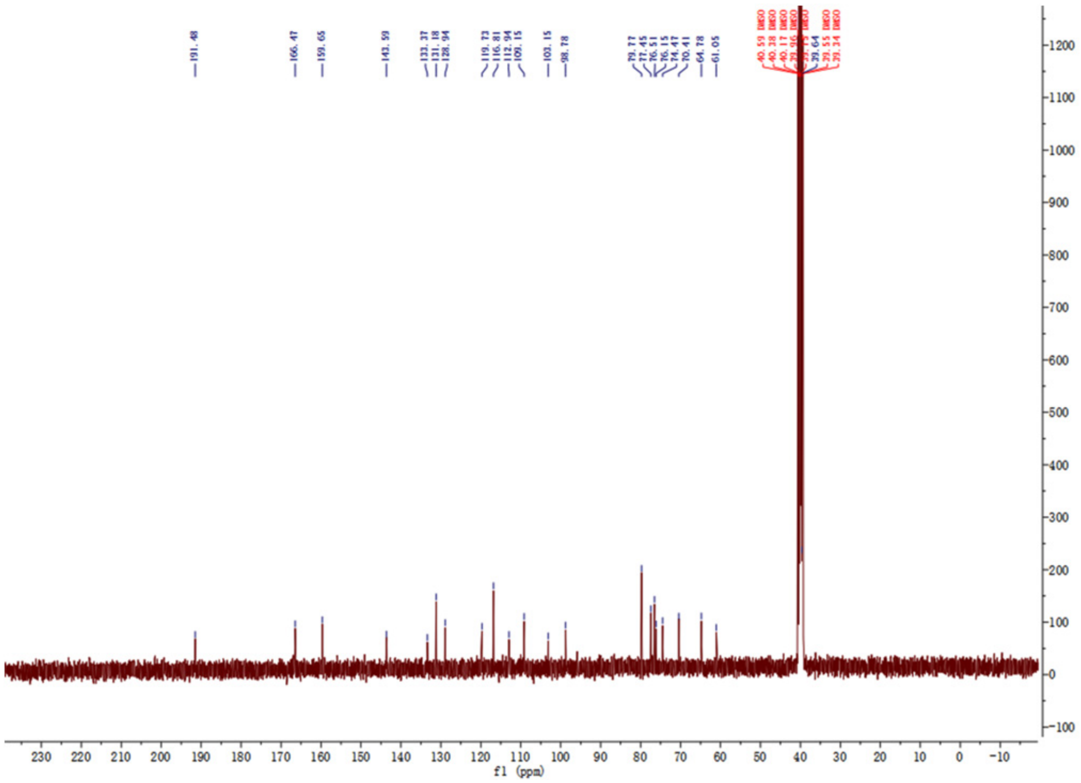

Figure S 13. **^{13}CNMR spectrum of Liquiritin apioside in DMSO**

Table S 1: Effects of six compounds from Flavone on activity of *β*-glucuronidase * (n = 3).

Compound	Concentration of the additives (mmol/L)						
	0.09	0.18	0.36	0.72	1.2	2.4	3
1	124.9±2.4	146.5±3.2	190.2±1.9	265.2±3.8	272.3±1.1	272.6±1.1	273.6±0.8
2	97.3±1.7	97.3±1.2	100.4±1.2	106.3±0.9	98.2±0.2	100.7±0.5	101.4±1.1
3	98.7±0.7	102.5±0.6	101.9±1.1	99.5±0.5	103.0±1.0	100.5±0.6	100.5±1.6
4	101.8±0.3	100.6±0.5	96.5±0.9	94.4±0.8	100.7±0.4	99.0±0.2	103.1±1.7
5	101.8±.03	97.8±0.3	97.5±0.7	101.7±0.6	100.5±0.6	100.2±0.7	102.3±2.0
6	106.7±1.2	97.7±1.3	105.4±0.9	101.9±1.1	100.4±0.8	105.1±1.5	101.3±1.0

*the activity in the *β*-glucuronidase solution (with 2.4 mmol/L of GL) without adding compound **1-6** was regarded as 100%. The reaction was conducted at 170 rpm and 40 °C for 2 h. One activity unit (U) of the *β*-glucuronidase enzyme was defined as the amount of enzyme capable of releasing 1 μmol GAMG per minute under the above conditions.
